# Lipid Accumulation in *Blastocystis* Increases Cell Damage in Co-Cultured Cells

**DOI:** 10.3390/microorganisms11061582

**Published:** 2023-06-14

**Authors:** Chen-Chieh Liao, Chun-Hsien Chen, Jyh-Wei Shin, Wei-Chen Lin, Chien-Chin Chen, Chun-Ting Chu

**Affiliations:** 1 Department of Parasitology, Institute of Microbiology and Immunology, College of Medicine, National Cheng Kung University, Tainan 701, Taiwangreatwall91983@gmail.com (C.-H.C.); wcnikelin@mail.ncku.edu.tw (W.-C.L.); 2 Department of Physiology, College of Medicine, National Cheng Kung University, Tainan 701, Taiwan; 3 Department of Microbiology and Immunology, College of Medicine, National Cheng Kung University, Tainan 701, Taiwan; 4 Institute of Basic Medical Sciences, College of Medicine, National Cheng Kung University, Tainan 701, Taiwan; 5 Department of Pathology, Ditmanson Medical Foundation Chia-Yi Christian Hospital, Chiayi 600, Taiwan; 6 Department of Cosmetic Science, Chia Nan University of Pharmacy and Science, Tainan 717, Taiwan; 7 Department of Biotechnology and Bioindustry Sciences, College of Bioscience and Biotechnology, National Cheng Kung University, Tainan 701, Taiwan; 8 Rong Hsing Research Center for Translational Medicine, National Chung Hsing University, Taichung 402, Taiwan; 9 Division of ColoRectal Surgery, Department of Surgery, Ditmanson Medical Foundation Chia-Yi Christian Hospital, Chiayi 600, Taiwan

**Keywords:** *Blastocystis* ST7-B, lipid, pathogenesis, Caco-2 cell

## Abstract

*Blastocystis hominis* is an intestinal protozoan that is often neglected, despite causing abdominal pain and diarrhea. Previous research has demonstrated that lipids can be synthesized by *B. hominis* or can accumulate in growth medium, but their function and mechanisms in the pathogenesis of *Blastocystis* remain unclear. Our study found that lipid-rich *Blastocystis* ST7-B can increase inflammation and disrupt Caco-2 cells more than the same parasite without the lipovenoes supplement. Additionally, the cysteine protease of *Blastocystis*, a virulence factor, is upregulated and has higher activity in lipid-rich *Blastocystis*. In order to better understand the effects of lipids on *Blastocystis* pathogenesis, we treated lipid-lowering pravastatin during *Blastocystis* ST7-B culturing with a lipovenoes supplement, which decreased the lipid levels of the *Blastocystis* and reduced the *Blastocystis*-induced inflammation and cell disruption of Caco-2 cells. We also analyzed the fatty acid composition and possible synthesis pathway in *Blastocystis* ST7-B, finding significantly higher ratios of arachidonic acid, oleic acid, and palmitic acid than in the other lipid components in lipid-rich *Blastocystis* ST7-B. These results suggest that lipids play a significant role in the pathogenesis of *Blastocystis* and provide important information on the molecular mechanisms of and potential treatments for *Blastocystis* infection.

## 1. Introduction

*Blastocystis* spp. is a single-celled eukaryote that is commonly found in the intestinal tracts of various animals and humans [1]. *Blastocystis* infection has a prevalence that ranges from 1.1% to 16.0% in developed countries such as Singapore, Japan, and Taiwan [2,3,4], and it can be as high as 60.0% in developing countries, including Senegal, Cuba, Brazil, and Argentina [5,6,7,8]. The forms of blastocystosis include common gastrointestinal symptoms, such as abdominal pain, vomiting, and bloating, as well as mucous and watery diarrhea [9]. Recently, irritable bowel syndrome (IBS), a functional gastrointestinal disorder that produces pain or discomfort in the abdomen and changes the frequency of bowel movements [10], has been frequently associated with *Blastocystis hominis* infection (blastocystosis) [11]. Previous studies that have compared various gastrointestinal symptoms in patients with other gastrointestinal disorders involving infection with *Blastocystis* spp. have shown that *Blastocystis* is significantly present in IBS patients [12,13]. 

Gastrointestinal disorders, such as celiac disease, IBS, and inflammatory bowel disease, are reported to be associated with a dysfunction of the epithelial barrier, which is mainly regulated by tight junctions [14]. Proteases are known to modulate the epithelium, inducing the disruption of tight epithelial junction complexes and causing subsequent barrier dysfunction [15]. The recruitment of cytokines, such as IL-1, IL-6, and IL-8, to specific sites of infection forms part of the inflammatory process, which can lead to the influx of inflammatory cells into the intestinal mucosa, causing gastrointestinal disturbances and tissue damage. Among these inflammatory cytokines, previous studies have demonstrated that a higher expression of IL-8 correlates with *Blastocystis*-induced cell damage. Puthia et al. showed that the cysteine proteases secreted by *B. ratti* WR1 could modulate IL-8 gene expression in human colonic epithelial cells. Additionally, *Blastocystis* cysteine proteases can activate IL-8 gene expression in human colonic epithelial cells via NF-κB activation [16]. The increase in intestinal permeability in blastocystosis is due to the reorganization of tight intestinal epithelial junctions mediated by cysteine proteases [17,18,19]. 

Mounting evidence suggests that lipid droplets play a crucial role in the fundamental mechanisms of host–pathogen interactions, including cell signaling and immunity [20]. Although the molecular mechanisms governing lipid droplet biogenesis have not been fully characterized, previous studies have shown that parasite lipid droplets compartmentalize eicosanoid synthesis, which is involved in parasite physiology and escape mechanisms [21,22]. For example, infecting erythrocytes from the malaria parasite *Plasmodium falciparum* induces lipid droplet formation within the parasite. Lipids that accumulate in the vacuoles of *Plasmodium falciparum* can increase parasite survival via their involvement in heme detoxification [23,24]. Proteomic studies have shown that lipid droplets are not simply cellular inclusions for neutral lipid storage but are rather functional cellular organelles [25,26]. Yoshikawa et al. reported that the localization of lipids produced by *B. hominis* was frequently observed in the cytoplasm and accumulated in the central vacuole, but the function of this is not well known [27]. In addition, previous studies have shown that lipids can be synthesized by *B. hominis* or can accumulate in a growth medium [28].

Statins are commonly prescribed as lipid-lowering agents because they act as a rate-limiting enzyme in the cholesterol biosynthesis pathway [29] and can decrease the viability of *Plasmodium falciparum* through the inhibition of the proteasome [30]. In a previous study, Tan et al. demonstrated the potential role of statins as anti-*Blastocystis* agents that modulate the barrier function of the intestinal epithelium and also prevent *Blastocystis*-induced barrier dysfunction in Caco2 cells [18]. Pravastatin is a first-generation statin in clinical use for its lipid-lowering effects [31]. Here, we investigate whether pravastatin can be used to reduce lipid storage in *Blastocystis* and produce a parasite-induced epithelial cytopathic effect.

In this study, our aim was to gain a better understanding of the relationship between lipid accumulation and the pathogenicity of ST7-B. We established an LR ST7-B strain and compared it to the normal strain, investigating the effects of pravastatin treatment on this protozoan. Additionally, we used Caco2 cells as a model to examine whether LR ST7-B induces a greater cytopathic effect during co-incubation. The results of this study may shed light on the interplay between lipids and pathogenesis in *Blastocystis* ST7-B and provide insights into potential therapeutic approaches for treating *Blastocystis* infection. 

## 2. Material and Methods

### 2.1. Blastocystis hominis and Caco-2 Cell Culture

The axenic *Blastocystis* subtype 7 isolate B was a gift from Prof. Kevin SW Tan. The *Blastocystis* ST7-B used in this study was isolated from a symptomatic patient at the Singapore General Hospital and classified as a subtype-7 isolate according to the Stensvold classification scheme [2,32]. In brief, the normal *Blastocystis* subtype 7 isolate B (ST7-B) was maintained in 8 mL of pre-reduced Iscove’s modified Dulbecco’s medium (IMDM) (Gibco) containing 10% horse serum (Gibco) in anaerobic jars at 37 °C. The lipid-rich *Blastocystis* ST7-B (LR ST7-B) parasites were grown in IMDM with a lipovenoes (Fresinius-Kabi) supplement (1%) culture for 72 h. The parasites were then washed in PBS before co-culture with Caco-2 cells. The number of viable cells was determined via trypan blue exclusion hemocytometer counts. Unsynchronized mid-logarithmic phase parasites with more than 98% viable cells were harvested for the construction of a cDNA library and proteomics study. 

The human intestinal epithelial cell line Caco-2 (ATCC^®^ HTB-37™) was a gift from the cell bank of the College of Medicine, National Cheng Kung University (NCKU), Taiwan. Caco-2 cells were used to study whether *Blastocystis* affects cell damage and gene expression in host cells. The Caco-2 cells were cultured in 30 or 100 mm^2^ cell culture dishes in an IMDM medium supplemented with 10% heat-inactivated FBS and antibiotics (50 μg/mL streptomycin and 100 U/mL penicillin). 

### 2.2. Assay of Cytopathic Effects (CPE)

Caco-2 cells were seeded at 5 × 10^5^ in a 24-well plate for 48 h. Caco-2 cells were then co-cultured with normal *Blastocystis* ST7-B and LR *Blastocystis* ST7-B at an MOI (multiplicity of infection) of 10. After co-incubation for 48 h, the cells were washed with PBS, paraformaldehyde-fixed, and then stained with Giemsa stain (Merck, Darmstadt, Germany). The quantitation of the cells’ coverage area was determined using Image-Pro Plus analysis.

### 2.3. Lipid Staining of Blastocystis hominis

*Blastocystis* ST7-B lipids were stained with Sudan Black B (Merck, Darmstadt, Germany) for neutral lipids, Oil Red O (Sigma-Aldrich, Taufkirchen, Germany) for oil drops, and Nile Red for triglycerides (Sigma-Aldrich, Taufkirchen, Germany). To specifically detect lipids, the normal *B. hominis* ST7-B and LR *Blastocystis* ST7-B were fixed in paraformaldehyde at 4% for 15 min after 72 h of culture. The cells were then washed in PBS and stained with Sudan Black B at 37 °C for 2 h, Oil Red O for 15 min, and Nile Red for 20 min. The quantification of pixel density was performed in one panel per parasite. Images of the same magnification (×400) were analyzed quantitatively using Image-Pro Plus 6.0 software (Media Cybernetics, Rockville, MA, USA).

### 2.4. Total RNA Isolation and cDNA Synthesis of Blastocystis ST7-B

Total RNA was extracted from *Blastocystis* ST7-B via TRI Reagent^®^ (Molecular Research Center, Cincinnati, OH, USA). The RNA was extracted with the Direct-zol™ RNA MiniPrep kit (Zymo Research, Irvine, CA, USA) and stored at −80 °C. High-Capacity cDNA Reverse Transcription Kits (Applied Biosystems, Foster City, CA, USA) were used in this study.

### 2.5. Library Preparation for RNA-seq

The RNA samples from *Blastocystis* ST7 B were used to generate sequence reads via the Illumina platform, giving more than 16 million sequence reads via the Molecular Chang Gung Medicine Research Center.

### 2.6. Quantitative Reverse Transcription PCR (RT-qPCR) Assay

The gene expression levels of the glycerol-3-phosphate dehydrogenase (GPDH) and glucose-6-phosphate dehydrogenase (G6PD) genes of *Blastocystis* ST7-B with or without the lipovenoes supplement were determined via qRT-PCR analysis. The GPDH forward primer used was 5′ GCGAACCCATGAAATCTGGC3′, and the reverse primer was 5′-TCCGACACTTTCCGATTGGG-3′. The G6PD forward primer used was 5′-TTCGGTCGCGACTATGACAG-3′, and the reverse primer was 5′-GTTCGAGAACCGAGTCACCA-3′. The SSU rRNA gene used BL18SPPF1 (5′-AGTAGTCATACGCTCGTCTCAAA-3′) and BL18SR2PP (5′-TCTTCGTTACCCGTTACTGC-3′) [33]. qPCR was performed using GoTaq^®^ qPCR Master Mix (Promega Corporation, Madison, WI, USA) via StepOne Real-Time PCR Systems (Thermo Scientific, Waltham, MA, USA). The following cycle conditions were used: 95 °C for 10 min, followed by 40 cycles at 95 °C for 30 s and 60 °C for 1 min. CT values were first normalized to the SSU rRNA gene and then to cells cultured in the media only.

The examination of IL-8 and cellular retinoic acid binding protein 2 (CRABP2) in Caco-2 was performed after co-incubation with *Blastocystis* ST7-B. qPCR was performed, as previously described [34]. Briefly, the 20 μL PCR mixtures contained 1 μg of reverse transcription product, master mix (Ampliqon), and 0.5 μM forward and reverse primers. The CRABP2 forward primer used was “5-GCTGAGGAAGATTGCTGTGG-3”, and the reverse primer was “5-AATTCTCTGGTCCAGGAGGT-3”. The IL-8 forward primer used was “5-ATGACTTCCAAGCTGGCCGTGGCT-3”, and the reverse primer was “5-TCTCAGCCCTCTTCAAAAACTTCTTC-3”. The following cycle conditions were used: 95 °C for 10 min, followed by 40 cycles at 95 °C for 30 s and 60 °C for 1 min. CT values were first normalized to GAPDH and then to cells cultured in the media only.

### 2.7. Protein Extraction for 2DE and Identification via MALDI-TOF-MS 

Caco-2 cells were harvested via centrifugation at 3000 rpm for 15 min and washed with saline three times. Lysis buffer (8 M urea, 4% CHAPS) was added to the cell pellet (1 mL lysis buffer for 1 × 10^8^ cells) for 5 min. After centrifuging at 13,000 rpm for 10 min at 4 °C, the supernatant was kept at −80 °C until use. A 2D clean-up kit (GE Healthcare, Chicago, IL, USA) was used to remove the impurities from the protein extract. The protein concentration was determined with a NanoDrop spectrophotometer (ND-1000, Thermo Scientific, Waltham, MA, USA). Samples were applied to 13 cm IPG gel strips (GE Healthcare) with a linear separation range of pH 4–7. The equilibrated IPG strips were separated on 10–15% SDS-PAGE gels and sealed with a solution of 0.5% (*w*/*v*) agarose containing a trace of bromophenol blue. The silver-stained 2DE gels were scanned and analyzed using Phoretix^TM^ 2D (Nonlinear Dynamics, Newcastle upon Tyne, UK) analysis software. The most highly expressed protein spots were selected and excised from the gels. Peptide extraction was performed twice for 15 min using sonication with an extraction buffer (100% ACN with 1% trifluoroacetic acid). Then, the peptides were analyzed using an Ultraflex MALDI-TOF Mass Spectrometer (Bruker Daltonic, Billerica, MA, USA). An automated database search was performed using the BioTool protein analysis software (Bruker Daltonic, Billerica, MA, USA). The protein score was represented as −10 Log (P), where P is the probability that the observed match is a random event. A global MASCOT score greater than 40 was considered significant in the NCBI database.

### 2.8. Fatty Acids Analysis with Gas Chromatography Mass Spectrometry (GC-MS)

The dry parasite pellets were weighed to determine the biomass (cell dry weight per unit volume, g/L) of the *Blastocystis* ST7-B culture. The fatty acid composition and content of the dry biomass of *Blastocystis* ST7-B were determined according to the protocols established in the Chen YM lab’s previous investigation. The samples underwent saponification/esterification reactions, which are chemical processes that convert fatty acids into their corresponding methyl esters (FAMEs). This involved mixing the samples with a 0.5 N NaOH methanol solution, heating them in a 90 °C water bath for 15 min, cooling them to room temperature, and then adding 0.7 N HCl in methanol and a 14% boron trifluoride methanol solution (Sigma-Aldrich, St. Louis, MO, USA). The samples were heated again in a 90 °C water bath for 15 min and then cooled. Next, 3 mL of saturated NaCl aqueous solution and 2 mL of n-hexane were added, and the sample was mixed well. The upper liquid layer was then transferred to a 4 mL amber vial, dried with a nitrogen stream, sealed with Parafilm^®^, and stored at −20 °C until analysis could be performed. The FAMEs were analyzed using gas chromatography with mass spectrometry. A CP-380 gas chromatography machine equipped with a 320 single-quadrupole mass spectrometer (Varian, Palo Alto, CA, USA) and a Supelco SP-2380 capillary column (30 m × 0.25 mm i.d.; Sigma-Aldrich, St. Louis, MO, USA) were used. The injector and interface temperatures were set at 250 °C and 270 °C, respectively. The column temperature was raised from 50 °C to 150 °C in increments of 15 °C/min and then from 150 °C to 250 °C in increments of 3 °C/min. The linear velocity of the carrier gas was 38.0 cm/min. The methyl esters prepared from fatty acids, including C20:2n − 6, C20:4n − 6, C22:2n − 6, C22:3n − 3, and C22:5n − 6 and a FAME standard mixture (Supelco 18919-1AMP) were all purchased from Sigma-Aldrich and used as standards for identifying fatty acids in the samples. The fatty acids were quantified according to their peak areas relative to the C19:0 fatty acid internal standard and expressed as a percentage of total fatty acid content [35].

### 2.9. Azocasein Assay for Cysteine Protease Activity

Parasite protease activity was determined via an azocasein assay, as described previously [36,37]. Different numbers of parasites (1 × 10^6^, 2 × 10^6^, 4 × 10^6^, 8 × 10^6^, and 1 × 10^7^) were harvested from the culture after 72 h and were washed twice in phosphate-buffered saline (PBS) (pH 7.4). Lysates were prepared by subjecting the *Blastocystis* to three freeze–thaw cycles using liquid nitrogen and a 37 °C water bath and subsequently storing them at a temperature of −80 °C. To activate protease activity, the parasite lysates were co-incubated at 37 °C for 10 min with 2 mM dithiothreitol (DTT) (Sigma). Azocasein, 100 μL of 5 mg/mL (Sigma) solution, was used in PBS (pH 7.4) and incubated with 100 μL parasite lysate at 37 °C for 1 h. A total of 300 μL of 10% trichloroacetic acid was added to stop the reaction, and the samples were then incubated on ice for 30 min. After centrifugation at 5000× *g* for 5 min at room temperature, the undigested pellets were removed, and the resultant supernatant was transferred to a new tube containing 500 μL of 525 mM NaOH. The negative controls using PBS and lysates were boiled for 15 min at 90 °C to inactivate the proteases. The positive control used 100 μL trypsin (2.5 mg/mL). Absorbance was determined with a spectrophotometer at 442 nm. The amount of enzyme that produced an increase of 0.01 OD units per hour was defined as one azocasein unit [36].

## 3. Results

### 3.1. Lipid Accumulation in ST7-B

**Figure 1:**
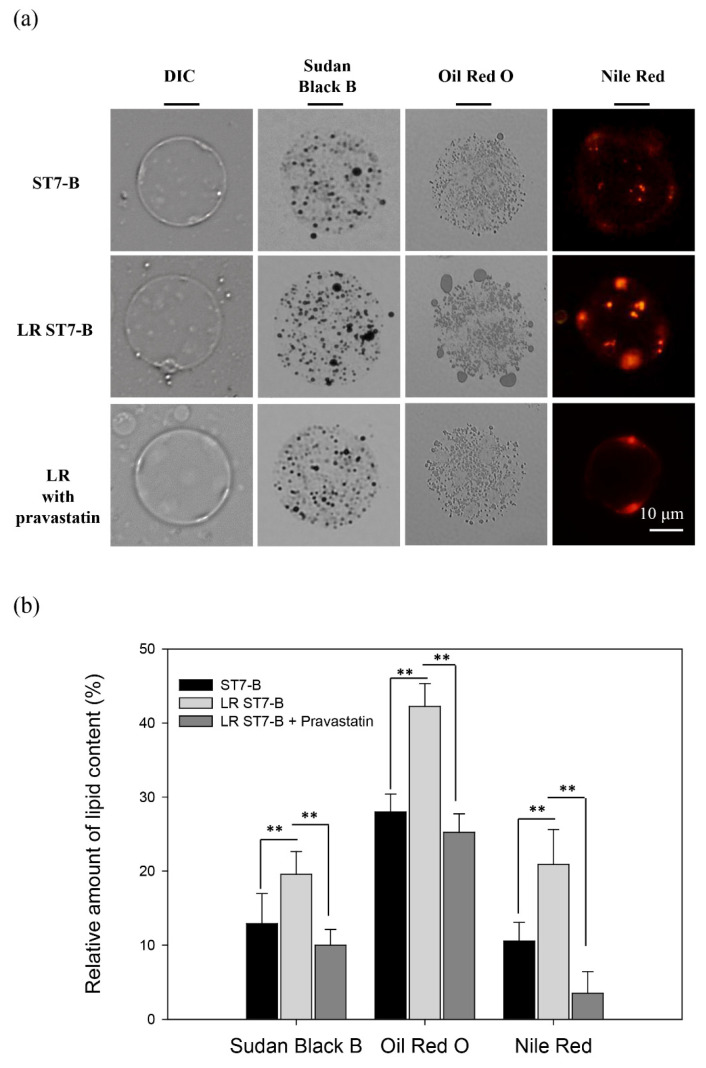
Lipid accumulation in *Blastocystis* ST7-B. (**a**) The different lipid stains of *Blastocystis* ST7-B; Sudan Black B for neutral lipid; Oil Red O for oil drop; Nile Red for triglycerides. Pravastatin reduced the triglycerides of *Blastocystis* ST7-B. (**b**) The quantifying of the lipid content in *Blastocystis* ST-7. N = 50, ** *p* < 0.01.

In a previous study, Yoshikawa et al. reported the presence of lipids in *B. hominis* [27]. We analyzed the lipids in *Blastocystis* ST-7 using a combination of stains: Sudan Black B for phospholipids, sterols, and neutral triglycerides; Oil Red O for neutral lipids and lipoproteins; and Nile Red for neutral lipids, including triglycerides. In order to investigate the effect of extracellular lipids on ST7-B lipid accumulation, we supplemented the medium with lipovenoes, which is rich in essential fatty acids, to induce lipid-rich ST7-B (LR ST7-B). Our findings showed that ST7-B could accumulate lipids, and LR ST7-B exhibited a significant increase in lipid accumulation upon lipovenoes supplementation (Figure 1a). Moreover, the quantification of the relative amounts of Sudan Black B, Oil Red O, and Nile Red showed a significant increase in LR ST7-B (up to a 1.51-, 1.51-, and 1.98-fold change, respectively, *p* < 0.05) (Figure 1b). TGs are one of the major components of lipovenoes, and our results indicate that supplementation with extracellular lipids significantly enhances their accumulation in ST7-B.

### 3.2. LR ST7-B Induced Significantly Higher Cell Inflammation and Cell Disruption in Caco-2 Cells

**Figure 2:**
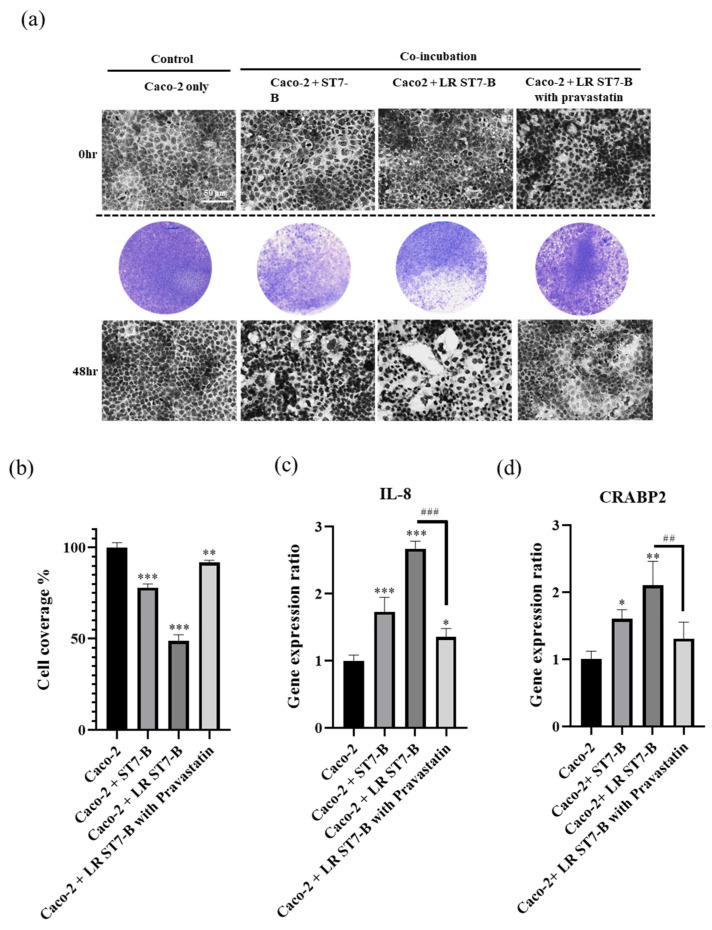
LR ST7-B induced significantly greater cell damage in Caco-2 cells. (**a**) Evaluation of the cytopathic effect of Caco-2 cells co-cultured with *Blastocystis* for 48 h via CPE assay. (**b**) Quantification of the coverage area of Caco-2 cells co-incubated with *Blastocystis* for 48 h. (**c**) IL-8 and (**d**) CRABP2 expression of Caco-2 cells co-incubated with *Blastocystis* for 48 h. Scale bar =100 um. * Compare the mean of each column with the mean of Caco-2; # Compare the mean of each column with the mean of Caco-2 + LR ST7-B with pravastatin. * *p* < 0.05, ** *p* < 0.01, *** *p* <0.01, ^##^ *p* <0.01, ^###^ *p* <0.001. The results were means ± SD of three independent experiments.

In order to simulate the host–pathogen interaction and detect the relationship between lipid modulation and the pathogenicity of *Blastocystis*, we used Caco-2 cells as a model to co-incubate with ST7-B in normal and lipid-rich conditions. The LR ST7-B induced significant cell disruption in the Caco-2 cells after co-incubation for 48 h using a CPE assay and time-dependent microscopy (Figure 2a,b) (*p* < 0.05). In our previous study, IL-8 and CRABP2 were used as pathogenesis markers for epithelial cells with *Blastocystis* co-incubation [38]. In normal conditions, the results were similar to our previous study: *Blastocystis* ST7-B induced IL-8 and CRABP2 expression in the Caco2 cells after co-incubation. After 48 h of co-incubation, both the IL-8 and CRABP2 levels in the Caco-2 cells co-incubated with LR ST7-B were significantly higher than those co-incubated with ST7-B (Figure 2c) (*p* < 0.05). LR ST7-B induced a severe cytopathic effect in the Caco-2 cells. 

### 3.3. Cysteine Protease Are Upregulated in LR ST7-B

**Figure 3:**
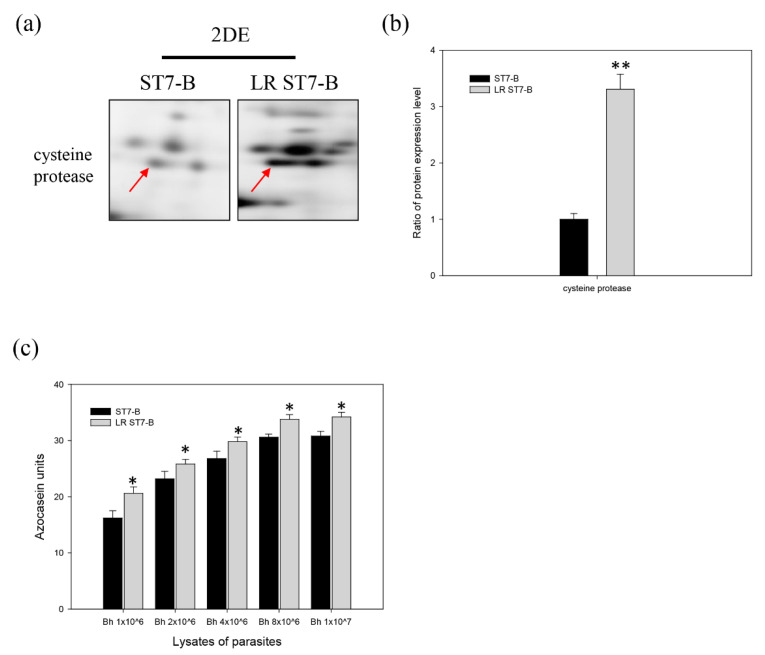
LR *Blastocystis* ST-7 upregulated cysteine protease expression. (**a**) The cysteine protease expression was upregulated in LR ST7-B, observed via 2DE analysis. (Red arrow: the 2D spot of cysteine protease) (**b**) Quantification of the cysteine protease expression of ST7-B and LR ST7-B. (**c**) The quantitative determination of the protease activity of *Blastocystis* ST7-B. Protease activity was determined with azocasein as a substrate. Lysates from 1 × 10^6^ to 1 × 10^7^ parasites were used for analysis. * *p* < 0.05, ** *p* < 0.01. The results were means ± SD of three independent experiments.

From the protease profile of *Blastocystis* ST7-B, the majority of proteases were cysteine proteases [37]. Cysteine proteases are putative virulence factors in *Blastocystis* and play an important role in inducing tight junction rearrangement and barrier dysfunction [18]. Here, the total protease activity of *Blastocystis* ST7-B was found to be significantly upregulated in a parasite-concentration-dependent manner. The protease activity of LR ST7-B was higher than in the normal ST7-B (Figure 3a). Based on a 2D electrophoresis analysis, cysteine protease was significantly upregulated in LR ST7-B (Figure 3b,c). (*p* < 0.05).

### 3.4. Pravastatin Lowered LR ST7-B-Induced Cell Inflammation and Reduced the Disruption of Caco-2 Cells

Pravastatin, which is prescribed as a lipid-lowering agent, can lower cholesterol levels [29]. In a previous study, Tan et al. also showed that statins act as an anti-*Blastocystis* agent to prevent barrier dysfunction in the intestinal epithelium [18]. Pravastatin was used as a lipid-lowering drug in this study and as a treatment for lipid accumulation for LR ST7-B co-incubated with Caco-2 cells. Our results using Nile Red staining showed that pravastatin can significantly decrease TG content by 50% in LR ST7-B (Figure 1). The CPE assay showed that pravastatin can also significantly reduce cell disruption, and the expression of both IL-8 and CRABP2 lowered the level of Caco-2 cells co-incubated with LR ST7-B (up to a 0.51-fold and 0.62-fold change, respectively, *p* < 0.05) (Figure 2).

### 3.5. Pravastatin Reduced the Viability of LR ST7-B

**Figure 4:**
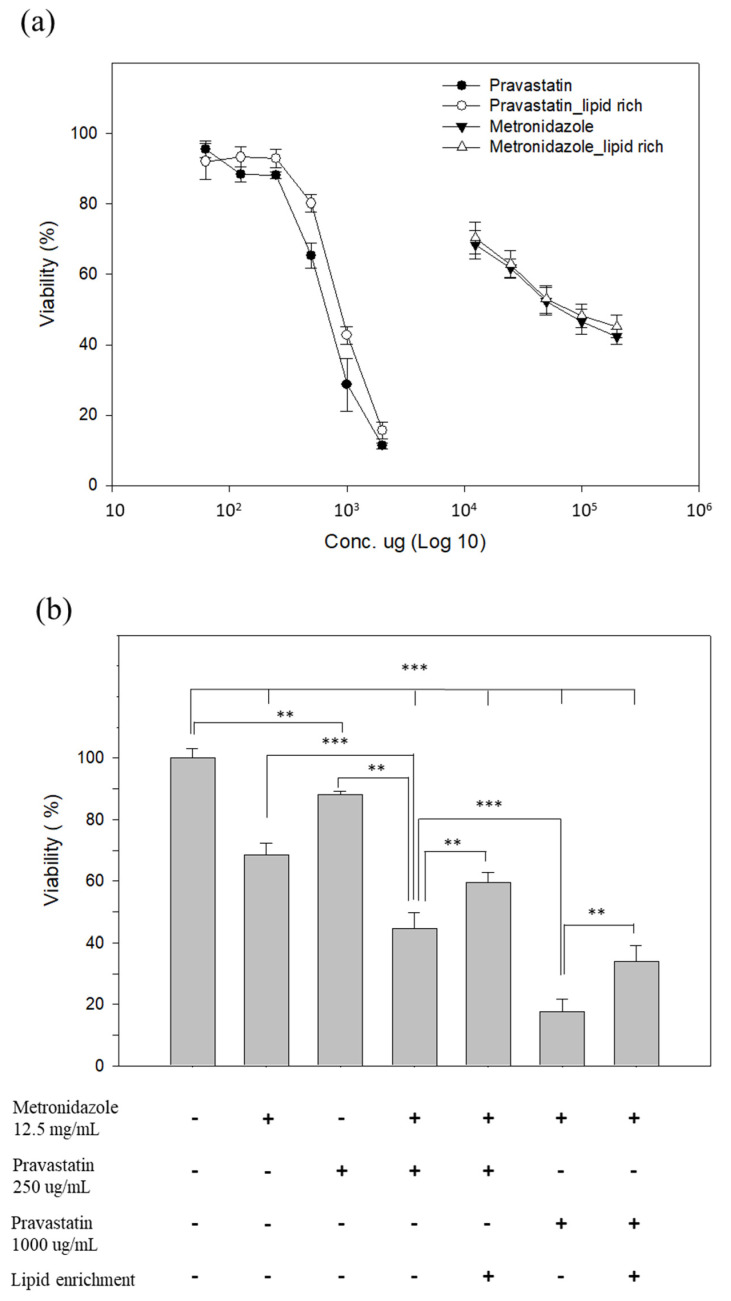
Drug treatment effect on pravastatin against *Blastocystis*. (**a**) Evaluation of the viability of *Blastocystis* ST7-B with pravastatin treatment. *Blastocystis* ST7-B exhibited a decrease in viability 24 h after treatment with varying doses of pravastatin ranging between 80 and 2000 μg mL^−1^. The viability of ST7-B also decreased via metronidazole, ranging between 12.5 and 200 mg mL^−1^. (**b**) LR ST7-B had significantly higher drug tolerance with metronidazole and pravastatin treatment. ** *p* < 0.01, *** *p* < 0.001. The results are means ± SD of three independent experiments.

Several studies have reported that *Blastocystis* may persist despite treatment, possibly due to its ability to resist metronidazole [39,40]. In a previous study, statins could modulate the barrier function of the intestinal epithelium and also prevent *Blastocystis*-induced barrier dysfunction in Caco2 cells [18]. Here, we aimed to investigate the effect of pravastatin on the growth and drug sensitivity of *Blastocystis* ST7-B. The viability results showed that treatment of the parasite with pravastatin reduced parasite viability in a dose-dependent manner, with an IC-50 value of 664.89 ± 42.82 µg mL^−1^ (normal condition) and 868.56 ± 32.65 µg mL^−1^ (lipid-rich condition) (Figure 4a). Metronidazole is a first-line treatment for blastocystosis and a mainstay in the treatment of intestinal protozoa [9,41]. The survival rate for *Blastocystis* ST7-B treatment with metronidazole at 12.5 mg mL^−1^ is 68.41%. The survival rate results showed that metronidazole (12.5 mg mL^−1^) combined with two different concentrations of pravastatin is effective against *Blastocystis* ST7-B; a dosage of 250 µg mL^−1^ can reduce the survival rate from 68.41 to 44.66 in a normal environment, and from 68.41 to 59.66 in LR ST7-B; a dosage of 1000 µg mL^−1^ can reduce the survival rate to 17.5% in a normal environment and 33.86% in LR ST7-B (Figure 4b).

### 3.6. The Composition of Fatty Acid in Blastocystis ST7-B

**Figure 5:**
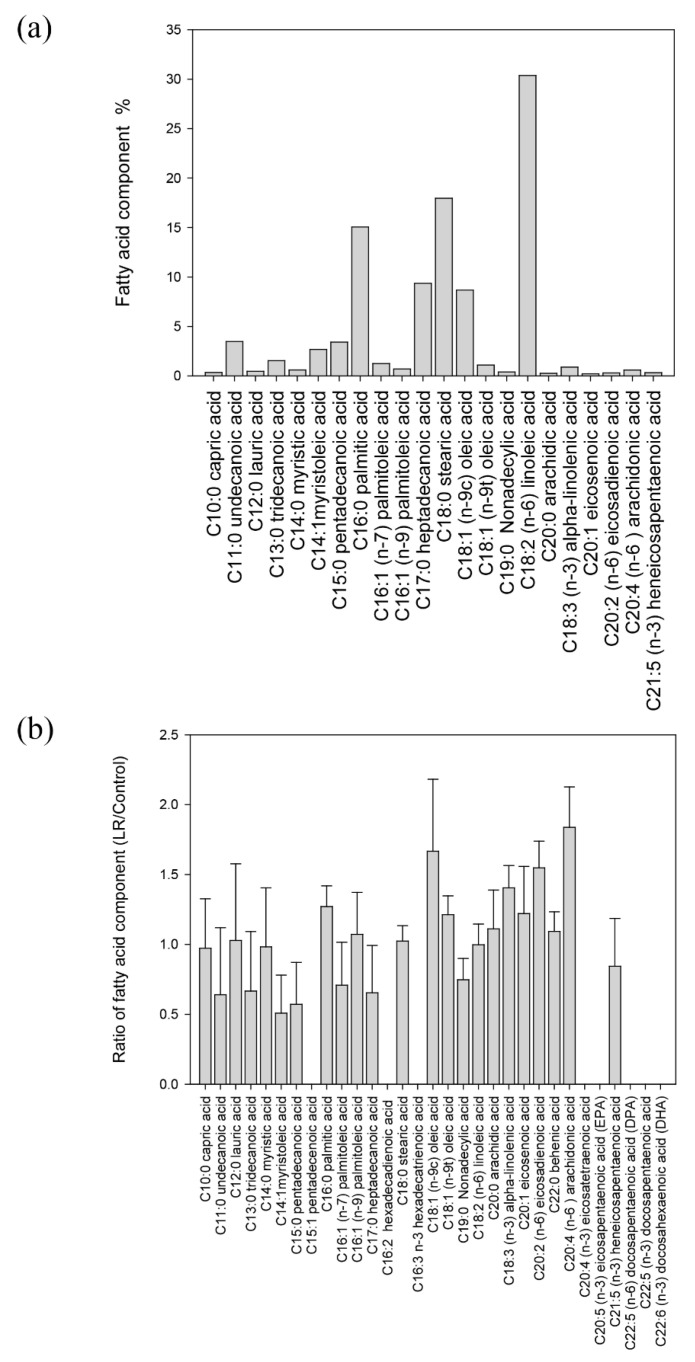
The fatty acid components of *Blastocystis* ST7-B. (**a**) The fatty acid composition was obtained from the whole-cell lipids of 1 × 10^7^/mL of ST7-B with GC-MS analysis. (**b**) The fatty acid composition of LR ST7-B compared to ST7-B.

In order to understand the fatty acid components of ST7-B, the GC-MS analysis results showed that there were 23 classes of fatty acids in ST7-B (Figure 5a). The main fatty acids were linoleic acid (30.4%), stearic acid (18.0%), and palmitic acid (15.1%). When comparing LR ST7-B and ST7-B, the compositions of arachidonic acid, oleic acid, and palmitic acid were significantly higher than the other lipid components in LR ST7-B (up to a 1.84-, 1.67, and 1.27-fold change, respectively, *p* < 0.05) (Figure 5b and Figure S1). 

### 3.7. GPDH and G6PD Were Upregulated in LR ST7-B by 2D Electrophoresis and Reverse Transcription PCR

**Figure 6:**
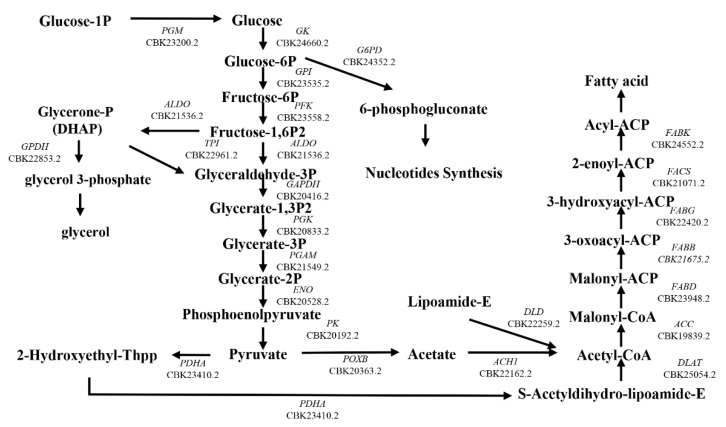
Reconstructed fatty acid synthesis pathway of ST7-B. The fatty acid synthesis pathway of ST7-B was mapped via the KEGG database based on Saccharomyces cerevisiae and *Entamoeba histolytica*.

**Figure 7:**
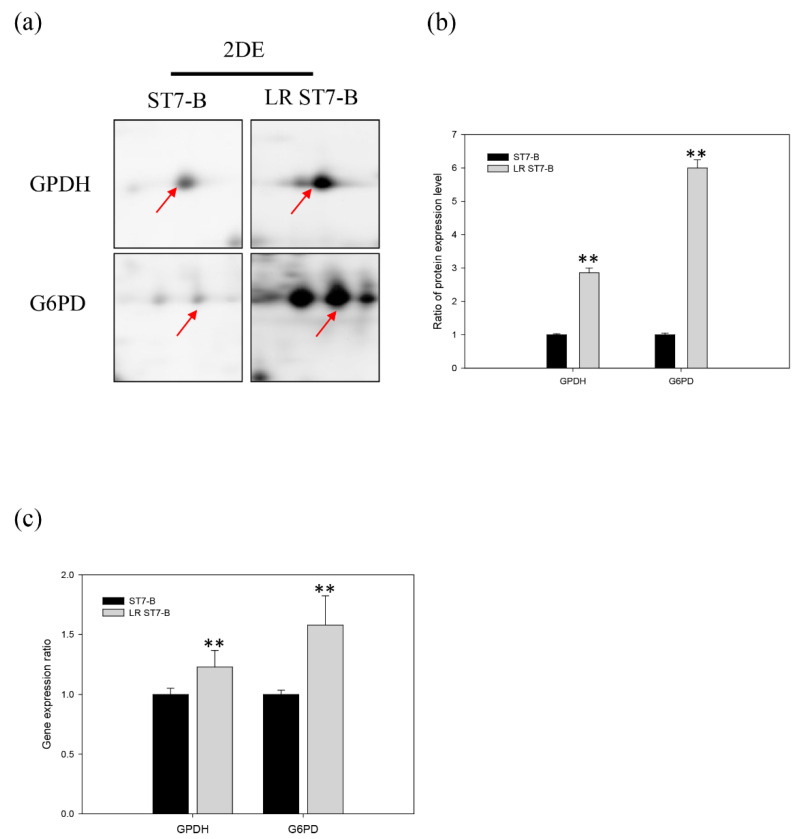
The expression of GPDH and G6PD of ST7-B and LR ST7-B. (**a**) The expression of proteins encoding the glycerol-3-phosphate dehydrogenase (GPDH) and glucose-6-phosphate 1-dehydrogenase (G6PD) of ST7-B and LR ST7-B, which were validated via 2DE analysis. (Red arrows: the 2D spot of GPDH or G6PD protein) (**b**) The quantification of GPDH and G6PD expression in ST7-B and LR ST7-B. (**c**) The gene expression of GPDH and G6PD in *Blastocystis* ST-7 via qPCR analysis. The SSU rRNA expression was used as a control. ** *p* < 0.01. The results are means ± SD of three independent experiments.

Keenan et al. found that *B. hominis* has the capacity to synthesize cellular lipids de novo but obtains cholesterol and cholesterol esters directly from the growth medium [28,42]. We used RNA-seq and 2D electrophoresis to identify the enzymes involved in the fatty acid synthesis pathway of *Blastocystis* ST7-B. We reconstructed the fatty acid synthesis pathway for 25 genes of ST7-B (Figure 6, Table S1). The 2D electrophoresis analysis revealed that LR ST7-B had significantly upregulated two proteins, glycerol-3-phosphate dehydrogenase (GPDH) and glucose-6-phosphate dehydrogenase (G6PD), with up to a 2.86-fold and 6-fold change, respectively (Figure 7a,b) (*p* < 0.05). In order to validate these results, we used qRT-PCR to measure the gene expression levels of GPDH and G6PD in *Blastocystis*. The qRT-PCR analysis confirmed the upregulation of both proteins in LR ST7-B, with up to a 1.2-fold and 1.6-fold change, respectively (Figure 7c). The results of our study indicate that supplementing extracellular lipids can increase the accumulation of lipids and activate the fatty acid synthesis pathway in *Blastocystis*.

## 4. Discussion

Lipids can be used as energy storage, signaling molecules, and pathogenic factors [43]. From previous reports and our results, we observed that *Blastocystis* accumulates lipids and forms lipid droplets (Figure 1). The lipid droplets are involved in different biological functions and contain not only lipids but also many proteins, such as the PAT family of proteins, including PERILIPIN, ADRP, and TIP47 [44]. *B. hominis* has the ability to synthesize lipids and acquire them from a culture environment via endocytosis [28,42]. Our study revealed that the predominant lipids in the central vacuole of ST7-B are TGs. Moreover, we observed that extracellular lipid supplementation leads to a significant increase in lipid accumulation within *Blastocystis*. GPDH and G6PD, which are involved in glycolysis, electron transport, glycerophospholipids metabolism, the hyperosmotic stress response, and anti-oxidative damage, play important roles in *Giardia* and *Malaria* [45,46]. Based on our results, we found the extra lipid enhanced the expression of GPDH and G6PD in *Blastocystis*, which may also play an important role in *Blastocystis*-induced cell damage

There are studies that show the important role of the composition and function of lipid-rich organelles and pathogenic prokaryotes and lower eukaryotes [44]. The lipid droplets of intracellular parasitic protozoa and bacteria play dynamic roles and are involved in signaling and inflammatory mediator synthesis [47]. These lipid droplets formed by protozoa and bacteria are able to seize host lipids or control the lipid biosynthesis mechanism of the host [48]. In this study, we found that arachidonic acid significantly increased in lipid-rich *Blastocystis* ST-7 and that it mediates inflammatory reactions [49]. Recent studies have shown that the lipid droplets of *Trypanosoma cruzi* play a dynamic role, including the response to host–parasite interactions and inflammation mediators, and they are involved in arachidonic acid metabolism [47]. Thus, *Blastocystis* may, via arachidonic acid, increase the production of IL-8 and cause the inflammation of colonic epithelial cells during co-incubation and affect some pathogenicity in clinical conditions. 

Lipids can also induce intestinal gas retention in patients with IBS, and excess gas has been reported in persons with *Blastocystis* infection [50]. Lipids inhibit intestinal gas transit, and this mechanism is upregulated in patients with IBS. In recent years, IBS has been frequently associated with *Blastocystis* infection [11,51]. The accumulation and biosynthesis of lipids in *Blastocystis* also caused cell inflammation and the disruption of Caco-2 cells. These results were more significant for lipid-rich *Blastocystis*. Thus, extra-cellular lipids may provide better conditions for *Blastocysts* to grow and exhibit pathogenicity. This may be a primary reason why IBS is frequently associated with *Blastocystis* infection.

Cysteine proteases represent an important factor in parasite immunity evasion and cell and tissue invasion in many parasitic organisms and may modulate the activity of the protease-activated receptors found on the epithelial cell surfaces of the host [52]. Previous studies have shown mainly cysteine protease activity in ST7-B, like many other protozoan parasites [37,53]. Our results showed that extra-cellular lipid supplementation could trigger ST7-B to express higher levels of cysteine protease. IL-8 and CRABP2 were upregulated in Caco-2 cells after co-incubation. LR ST7-B caused significantly higher cell disruption and inflammation in Caco-2 cells. Thus, the more lipid supplement, the greater the cell damage of Caco-2 cells when co-incubated with *Blastocystis*. Intestinal barrier destruction is an important index of cell disruption during blastocystosis [18]. Microenvironment modulation affects blastocystosis through the disruption and inflammation of the intestinal barrier. These results may explain the relationship between blastocystosis and IBS patients.

Previous studies have shown that the role of cholesterol and lipids is important in the pathogenesis of parasitic infections such as *Entamoeba*, *Giardia,* and *trichomonas* [54]. Statins are HMG-CoA reductase inhibitors that lower the level of cholesterol and lipids; they also have a range of other effects, including the restoration of endothelial function and anti-inflammatory effects [55]. Our results show that pravastatin reduced the lipid storage and viability of LR ST7-B and could decrease the cytopathic effect and CRABP2 and IL-8 expression of Caco-2 cells co-incubated with LR ST7-B. Here, pravastatin could help to alleviate the pathogenicity of *Blastocystis*. A similar result was also observed by Tan et al., specifically the potential role of statins as modulators of intestinal epithelial barrier functioning, which prevent tight junction rearrangements and barrier dysfunction in the intestinal epithelium by inhibiting the Rho-kinase-mediated myosin-light-chain phosphorylation of the host [18]. 

Metronidazole is a well-known, standard clinical treatment for *Blastocystosis* and a mainstay in the treatment of intestinal protozoa [9,41]. A recent study showed the need to develop other therapy options for parasite infections; some clinical *Blastocystis* isolates decreased susceptibility to conventional antibiotic agents [56]. In this study, pravastatin, in combination with metronidazole, also demonstrated synergistic effects against *Blastocystis* ST-7. The pleiotropic effect of statins presents one potent treatment option for antibiotic-resistant blastocystosis.

## 5. Conclusions

Our studies focused on the relationship between pathogenic potential and the lipid accumulation and biosynthesis of *Blastocystis*. LR ST7-B induced a higher cytopathic effect in Caco-2 cells. These results show that lipids play an important role in *Blastocystis* infection, which may cause pathogenicity in patients. A higher lipid level can cause cysteine protease to increase the disruption and inflammation of Caco-2 cells co-incubated with *Blastocystis*. Furthermore, pravastatin can reduce the viability of *Blastocystis* ST-7 and lower the parasite-induced inflammation and disruption of Caco-2 cells. Here, we suggest the therapeutic potential of statins for blastocystosis. Pravastatin, in combination with metronidazole, also demonstrated synergistic effects that were more efficacious against blastocystosis. We anticipate that our study may help us to understand the function of lipids and molecular mechanisms in blastocystosis. Although the other mechanisms are yet to be clarified, our findings suggest that the lipids existing in *Blastocystis* or in its microenvironment may be important to its pathogenicity.

## Data Availability

The data presented in this study are available at a reasonable request from the corresponding author.

## Supplementary Materials

The following supporting information can be downloaded at: https://www.mdpi.com/article/10.3390/microorganisms11061582/s1, Figure S1: The fatty acid components of (a) ST7-B and (b) LR ST7-B via GC-MS analysis.; Table S1: The 25 genes of fatty acid synthesis pathway in *Blastocystis* ST7-B compared to different *protozoa.*
