# Supplementary material for: Lipid Accumulation in Blastocystis Increases Cell Damage in Co-Cultured Cells
Source: Microorganisms. 2023 Jun 14;11(6):1582. doi: 10.3390/microorganisms11061582 (PMC10305000; doi:10.3390/microorganisms11061582)
Supplement: Supplementary file 1 [file microorganisms-11-01582-s001.zip › microorganisms-2317112-supplementary.pdf]

Supplementary Materials

(a)

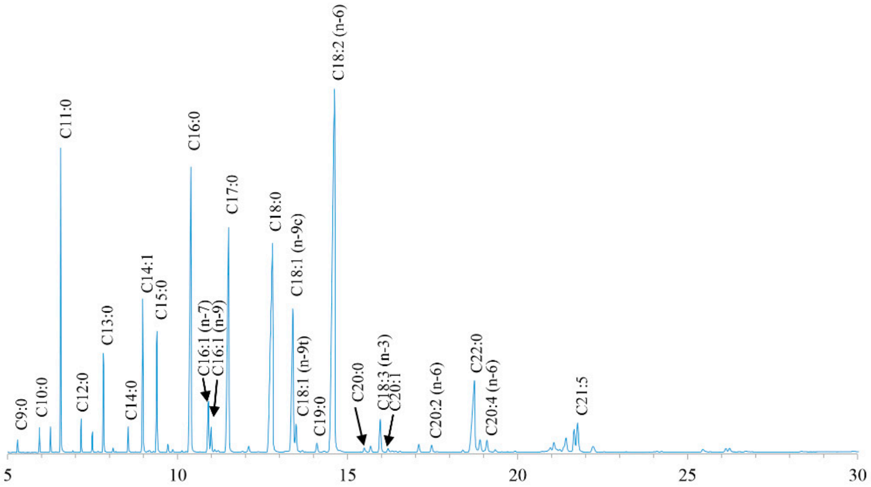

(b)

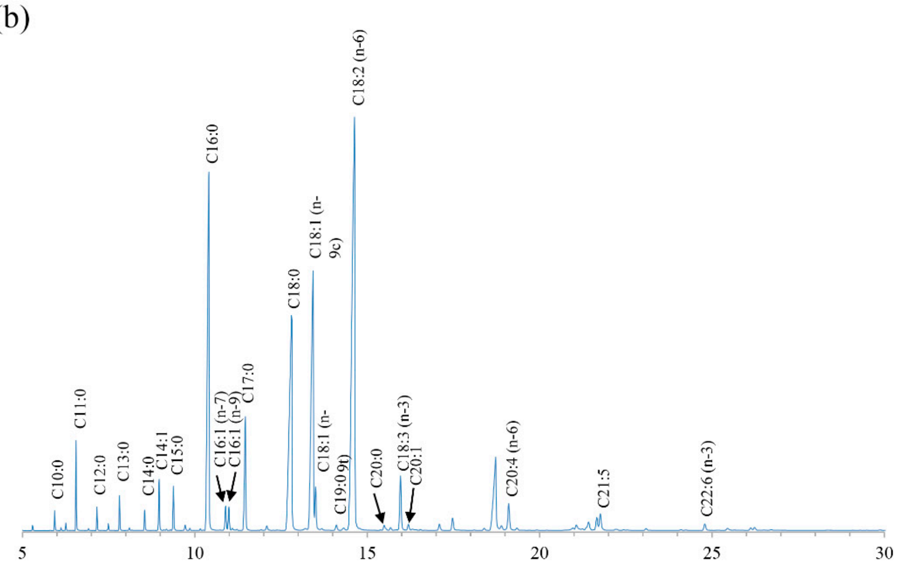

Figure S1. The fatty acid components of (a) ST7-B and (b) LR ST7-B via GC-MS analysis.

Table S1. The 25 genes of fatty acid synthesis pathway in *Blastocystis* ST7-B compared to different *protozoa*

accession number	enzyme	*Blastocystis ST-7*	*Amoeba*	Cryptosporidium	*Giardia lamblia*	*Plasmodium*	*Toxoplasma*	*Trichomonas vaginalis*	*Saccharomyces cerevisiae*
CBK23200.2	phosphoglucomutase	2.00E-180	3.00E-169	3.00E-04	9.00E-09	6.00E-156	1.00E-174	2.00E-131	3.00E-179
CBK24660.2	Glucokinase	6.00E-24	N	N	N	N	N	N	1.00E-26
CBK23535.2	Glucose-6-phosphate isomerase	0	3.00E-158	5.00E-23	6.00E-152	2.00E-177	3.00E-124	2.00E-123	0
CBK23558.2	6-phosphofructokinase 4	3.00E-100	3.00E-16	4.00E-13	3.00E-15	2.00E-15	8.00E-76	3.00E-10	3.00E-114
CBK21536.2	Fructose-bisphosphate aldolase	8.00E-121	N	2.00E-13	N	N	N	8.00E-102	5.00E-120
CBK20833.2	Phosphoglycerate kinase	1.00E-122	7.00E-80	1.00E-77	6.00E-83	7.00E-95	3.00E-85	2.00E-80	3.00E-121
CBK20416.2	Glyceraldehyde-3-phosphate dehydrogenase	8.00E-53	9.00E-47	9.00E-49	7.00E-39	1.00E-43	3.00E-36	5.00E-42	5.00E-52
CBK21549.2	2,3-bisphosphoglycerate-dependent phosphoglycerate mutase	3.00E-87	6.00E-70	0.004	9.00E-76	9.00E-77	8.00E-04	1.00E-61	2.00E-96
CBK20528.2	Enolase	3.00E-157	3.00E-137	2.00E-106	3.00E-151	7.00E-141	5.00E-130	5.00E-139	3.00E-164
CBK20192.2	Pyruvate kinase	3.00E-114	7.00E-109	7.00E-41	2.00E-103	5.00E-116	2.00E-71	2.00E-77	3.00E-128
CBK23410.2	Pyruvate dehydrogenase E1 component subunit alpha type II	7.00E-75	N	N	2.00E-29	6.00E-38	3.00E-60	4.00E-75	1.00E-180
CBK20363.2	Pyruvate dehydrogenase	0.00E+00	0.00E+00	0.00E+00	3.00E-11	8.00E-10	3.00E-45	1.00E-54	0.00E+00
CBK22162.2	Acetyl-CoA hydrolase	1.00E-175	N	N	N	N	N	3.00E-163	0
CBK22259.2	Pyruvate Dehydrogenase E3 (dihydrolipoamide dehydrogenase)	1.00E-75	2.00E-64	4.00E-38	9.00E-66	5.00E-66	5.00E-50	1.00E-59	5.00E-126
CBK25054.2	Pyruvate Dehydrogenase E2 (dihydrolipoamide acetyltransferase)	4.00E-76	5.00E-65	2.00E-38	1.00E-66	2.00E-66	3.00E-50	4.00E-60	1.00E-126
CBK19839.2	acetyl-CoA carboxylase	3.00E-43	6.00E-11	9.00E-06	1.00E-08	6.00E-11	2.00E-07	2.00E-12	3.00E-49
CBK23948.2	Malonyl CoA-acyl carrier protein transacylase	5.00E-19	3.00E-07	N	2.00E-05	4.00E-08	N	0.004	7.00E-21
CBK21675.2	3-oxoacyl-ACP synthase	4.00E-50	5.00E-13	N	2.00E-43	4.00E-40	4.00E-42	3.00E-35	3.00E-52
CBK22420.2	3-oxoacyl-ACP reductase	3.00E-07	N	N	N	N	N	N	1.00E-18
CBK21071.2	Fatty acyl-CoA synthetase A	4.00E-95	3.00E-76	3.00E-67	3.00E-81	2.00E-81	2.00E-72	1.00E-63	4.00E-106
CBK24552.2	Enoyl-[acyl-carrier-protein] reductase	2.00E-33	3.00E-21	7.00E-14	5.00E-12	3.00E-08	1.00E-12	5.00E-27	9.00E-38
CBK22853.2	Glycerol-3-phosphate dehydrogenase	3.00E-87	3.00E-64	N	1.00E-80	3.00E-85	4.00E-23	2.00E-76	1.00E-94
CBK21536.2	Fructose-bisphosphate aldolase class 2	8.00E-121	N	2.00E-13	N	N	N	8.00E-102	5.00E-120
CBK22961.2	Triosephosphate isomerase	2.00E-75	8.00E-68	2.00E-59	2.00E-62	5.00E-71	1.00E-60	2.00E-65	6.00E-78
CBK24352.2	Glucose-6-phosphate 1-dehydrogenase, cytoplasmic isoform	6E-148	N	2.00E-58	3.00E-100	4.00E-121	1.00E-126	3.00E-121	1.00E-146

N=none dected
